# Whole-Brain Exploratory Analysis of Functional Task Response Following Erythropoietin Treatment in Mood Disorders: A Supervised Machine Learning Approach

**DOI:** 10.3389/fnins.2019.01246

**Published:** 2019-11-20

**Authors:** Søren F. V. Nielsen, Kristoffer H. Madsen, Maj Vinberg, Lars V. Kessing, Hartwig R. Siebner, Kamilla W. Miskowiak

**Affiliations:** ^1^Copenhagen Affective Disorder Research Centre, Psychiatric Centre Copenhagen, Copenhagen University Hospital, Rigshospitalet, Copenhagen, Denmark; ^2^Department of Psychology, University of Copenhagen, Copenhagen, Denmark; ^3^Danish Research Centre for Magnetic Resonance, Centre for Functional and Diagnostic Imaging and Research, Copenhagen University Hospital Hvidovre, Hvidovre, Denmark; ^4^Section for Cognitive Systems, Department of Applied Mathematics and Computer Science, Technical University of Denmark, Kongens Lyngby, Denmark; ^5^Department of Neurology, Bispebjerg Hospital, Copenhagen University, Copenhagen, Denmark

**Keywords:** erythropoietin, functional magnetic resonance imaging, machine learning, mood disorders, cognitive dysfunction

## Abstract

A core symptom of mood disorders is cognitive impairment in attention, memory and executive functions. Erythropoietin (EPO) is a candidate treatment for cognitive impairment in unipolar and bipolar disorders (UD and BD) and modulates cognition-related neural activity across a fronto-temporo-parietal network. This report investigates predicting the pharmacological treatment from functional magnetic resonance imaging (fMRI) data using a supervised machine learning approach. A total of 84 patients with UD or BD were included in a randomized double-blind parallel-group study in which they received eight weekly infusions of either EPO (40 000 IU) or saline. Task fMRI data were collected before EPO/saline infusions started (baseline) and 6 weeks after last infusion (follow-up). During the scanning sessions, participants were given an n-back working memory and a picture encoding task. Linear classification models with different regularization techniques were used to predict treatment status from both cross-sectional data (at follow-up) and longitudinal data (difference between baseline and follow-up). For the n-back and picture encoding tasks, data were available and analyzed for 52 (EPO; *n* = 28, Saline; *n* = 24) and 59 patients (EPO; *n* = 31, Saline; *n* = 28), respectively. We found limited evidence that the classifiers used could predict treatment status at a reliable level of performance (≤60% accuracy) when tested using repeated cross-validation. There was no difference in using cross-sectional versus longitudinal data. Whole-brain multivariate decoding applied to pharmaco-fMRI in small to moderate samples seems to be suboptimal for exploring data driven neuronal treatment mechanisms.

## Introduction

Cognitive impairment in attention, memory and executive functions is a core symptom of unipolar disorder (UD) and bipolar disorder (BD) that often persists after clinical remission from mood symptoms (Bortolato et al., 2015; Kaser et al., 2017). However, there are no available effective treatments for these cognitive symptoms with well-understood neuronal mechanisms. Drug development for neuropsychiatric disorders is expensive and time-consuming, and biomarker models of the early drug effect are important (Borsook et al., 2013; Nathan et al., 2014). Functional magnetic resonance imaging (fMRI) of participants undergoing pharmacotherapy provide insight into treatment-related changes in cognition-relevant neural circuits and may thus aid the development of reliable biomarkers of treatment efficacy (Nathan et al., 2014). Application of fMRI in randomized controlled trials (RCTs) targeting cognition is therefore a key recommendation by the International Society for Bipolar Disorders (ISBD) Targeting Cognition Task Force (Miskowiak et al., 2017).

The growth factor erythropoietin (EPO) has been shown to be a promising pharmacological treatment of cognitive impairments across neuropsychiatric disorders including mood disorders (Ehrenreich et al., 2007a, b; Miskowiak et al., 2014a, b). In particular, EPO and its receptor are expressed in the brain and play and important role in neurodevelopment as well as in neuroprotection and neuroplasticity in preclinical models of acute neural damage and chronic neurodegenerative conditions (Sirén et al., 2009). The neuronal correlates of the beneficial effects of EPO on cognition in humans was investigated using fMRI in two parallel identical RCTs in UD and BD, respectively (Miskowiak et al., 2016a, b). Specifically, participants in the trials were given a working memory (WM) and picture encoding task during fMRI before and after EPO vs. saline treatment. Analysis of the changes in neuronal activity within task-positive neural networks indicated increased dorsal prefrontal and parietal activity across both tasks in participants given EPO vs. saline, which correlated with improved task performance. However, due to the hypothesis-driven approach, these reports rendered no insight into any unforeseen changes in the patterns of neural activity in response to EPO vs. saline. Furthermore, the extracted between-group differences in brain activity found in these studies do not necessarily provide any understanding of the models predictive capabilities in a held-out sample (Bzdok et al., 2018).

Machine learning methods have attracted great recent research interest because of their ability to identify variables in a given dataset that are relevant or irrelevant to an outcome of interest, while conventional statistical analyses rely on investigator specified variables of relevance to a particular analysis (Bzdok and Meyer-Lindenberg, 2018). A recent systematic review identified eight structural or fMRI studies investigating baseline neuroimaging features that informed algorithms predicting treatment efficacy in depression (Lee et al., 2018). Two studies found that predictors of treatment response to electroconvulsive therapy were baseline subgenual and cingulate volume (Redlich et al., 2016) and resting state connectivity in the dorsomedial PFC and ACC (van Waarde et al., 2014), respectively. Other studies found that treatment response to antidepressant drugs was predicted by baseline middle frontal and angular gyrus volume (Korgaonkar et al., 2015), white and gray matter volume (Liu et al., 2012), lower WM integrity in the salience network and lower FC in the dorsal default mode network (Patel et al., 2015) or neural activity during a WM task (Marquand et al., 2008). Finally, one study investigated baseline predictors of response to cognitive behavioural therapy (CBT) which revealed neural activity to emotional stimuli as significant predictors (Costafreda et al., 2009). However, no study to date has used machine learning algorithms to investigate the neural correlates of treatment effects using longitudinal data sets.

Expanding the mass-univariate analyses already carried out in Miskowiak et al. (2016a, b), we adopted a supervised machine learning approach to explore the predictive value of the fMRI data from the spatial n-back and picture encoding paradigms. We pooled data from our two parallel identical trials in treatment-resistant depression and BD for the present analysis because of the similar abnormalities in neural activity during WM and spatial memory encoding across unipolar and BDs (Miskowiak and Petersen, 2019) and similar effects of EPO on cognitive function and neural activity across these groups (Miskowiak et al., 2016a, b; Ott et al., 2016). We applied a whole-brain multivariate supervised machine learning approach inspired by Barron and colleagues (Barron et al., 2018) to investigate whether the administration of EPO can be predicted (reverse inference) by the fMRI blood oxygen level dependent (BOLD) response during these tasks after treatment completion (primary aim). Furthermore, we compare the predictive ability of the model when applied to the *cross-sectional* post-treatment data or when using the *longitudinal* change data (follow-up minus baseline). Intra-subject variability stemming from between-session sources of variance has an influence on the reliability of the fMRI task response (Lund et al., 2005; Gonzalez-Castillo et al., 2017). By comparing the cross-sectional data to the longitudinal data, we seeked insight into the role of intra-subject fMRI task response variability in drug trials.

We hypothesized that the whole-brain multivariate supervised machine learning analysis would be able to predict which patients had received EPO based on the post-treatment neural task responses. As an exploratory analysis, we also investigate whether treating the data as longitudinal vs. cross-sectionally yields a difference in classification performance.

## Materials and Methods

### Study Design and Participants

This report is based on the double-blind randomized placebo-controlled trial described in Miskowiak et al. (2014a, b) (clinicaltrials.gov, NCT00916552). Participants included in the report were between 18 and 65 years of age and had a diagnosis of either treatment resistant unipolar depression with moderate depression severity (Hamilton Depression Rating Scale 17-item scale (HAMD-17) ≥ 17) or BD in partial or full remission (HAMD-17 and Young Mania Rating Scale (YMRS) ≤ 14) with moderate to severe cognitive complaints. Trial participants received eight weekly intravenous infusions of EPO or saline (NaCl 0.9%) and underwent whole-brain fMRI at baseline (pre-infusions) and at week 14 (follow-up), 6 weeks after treatment completion when blood parameters had normalized in the EPO group. For more details on the study design, inclusion and exclusion criteria see Miskowiak et al. (2014a, b).

### Spatial n-Back Working Memory Task

The task was divided into blocks of three conditions with increasing WM load (0-back, 1-back, and 2-back). Within each condition, a yellow circle was shown for 300 ms in one of 25 locations distributed in a grid (5 × 5). The circle was followed by an empty grid for 1200 ms, and this was repeated 14 times per block. In 1-back and 2-back conditions, participants were instructed to press a button whenever the circle appeared at the same location as one trial or two trials back, respectively. During the 0-back condition, participants pressed a button whenever the circle appeared in one of the four grid corners. Each block had an average of three target trials and were presented successively five times for each condition, resulting in a total of 15 stimulus blocks. After each block an 8 s fixation cross was shown, and the total task length was 7 min 35 s.

### Explicit Picture Encoding Task

In the second paradigm administered during scanning, pictures were presented in blocks of 24 s interleaved with blocks of 24 s fixation crosses. Within each picture-block, six pictures were presented for three seconds each with a 1 s fixation cross in between. Participants were asked to look carefully at the pictures and memorize them as they would be asked to recall the pictures after then scan. After scanning, participants were given a free recall test. A matched parallel version of the task was administered at the follow-up scan to mitigate learning effects. The total task duration was 4 min and 50 s.

### fMRI Setup

The detailed fMRI protocol is described in Miskowiak et al. (2016a, b) and the following is a short summary. Whole-brain MRI was performed with a 3-T Siemens Trio MR scanner using an eight-channel head array coil at the Danish Research Centre for Magnetic Resonance. BOLD-sensitive fMRI involved a T2^∗^-weighted echo-planar imaging (EPI) sequence with an echo time (TE) of 30 ms, repetition time (TR) of 2.49 s and a flip angle of 20° to minimize physiological noise. For the spatial WM task, a total of 184 brain volumes were acquired in a single fMRI session, each consisting of 42 slices acquired in interleaved order with a slice thickness of 3 mm and a field of view (FOV) of 192 × 192 mm using a 64 × 64 acquisition matrix. For the explicit picture encoding task, a total of 117 brain volumes were collected. High-resolution 3D structural T1-weighted images were obtained after the first session of BOLD fMRI (TI = 800, TE = 3.04, TR = 1550 ms, flip angle 9°; 256 × 256 FOV; 192 slices).

### Preprocessing and Data Analysis

Preprocessing in FMRIB’s Software Library (FSL) package version 5.0.2.2^1^ included, B0 unwarping, motion correction using MCFLIRT, spatial smoothing with a FWHM 5 mm Gaussian kernel, high-pass filtering with cutoff at 1/100s and linear registration to standard space (MNI152). We ran a first-level analysis in FSL’s FEAT, where the standard and extended motion parameters from MCFLIRT were entered as nuisance regressors. Data from three participants were excluded due to signal loss which was apparent from the estimated mask. Contrast of parameter estimates (COPE) are obtained from FSL. No thresholding was applied to any of the COPE maps in the subsequent analyses. For the picture encoding task, the picture-related contrast (i.e., average signal during picture presentation) was used to examine encoding-related neural activity. For the spatial WM task, the 2-back minus 0-back contrast was used to explore the neural activity specifically involved in WM performance.

For studying longitudinal changes, we obtained contrast maps of change in neural response taking the COPE difference between baseline and follow-up scans for each participant separately. Both the cross-sectional and longitudinal changes COPES maps were normalized by dividing by the participant mean image. The normalized maps formed the input to the subsequent whole-brain machine learning method along with the label “EPO” or “Saline” according to the treatment received. The group mask (intersection) of the included subjects was constructed for each task separately, yielding 199602 and 199820 voxels for picture encoding and n-back task, respectively.

#### Shen Parcellation

To investigate the differences between using whole-brain cope maps and a parcellation, we use the Shen parcellation (Shen et al., 2013) inspired by the approach used by Barron et al. (2018). The features for the subsequent machine learning analysis were extracted by averaging the COPE maps in each parcel. For the cross-sectional analysis, we chose to map the Shen parcellation from standard space to each subjects native space, using the transformation estimated in the FSL pipeline. In the case of the longitudinal analysis, all COPE maps were already in MNI space and thus we just naturally used the parcellation in MNI. In both types of analyses, we remove parcels without any signal in them, i.e., parcels that are outside the mask estimated by FSL. This resulted in 231 parcels for both tasks.

### Whole-Brain Multivariate Decoding Using Logistic Regression

For decoding what treatment (EPO or Saline) the participant has received based on the neural response maps, *x*∈ℝ^*v*^, we use logistic regression. We model the probability of the treatment to participant *n* being EPO given *x*_*n*_, *p*(*y*_*n*_ = 1|*x*_*n*_), by

$$p\left(y_{n}=1|x_{n}\right)=\phi \left(w^{T}x_{n}+w_{0}\right)=\frac{1}{1+e^{-w^{T}\,x_{n}+w_{0}}}$$

in which *w* is the weight vector estimated during training and *w*_*0*_ is the intercept. Participants given the placebo treatment have *y*_*n*_ = 0. Since we have very few observations (*N*) compared to the number of voxels in the difference maps (*v*), we use a regularizer, θ(*w*,*w*_0_), to prevent overfitting such that the cost-function of model becomes,

$$E\,\left(w,w_{0}\right)=-\sum_{n=1}^{N}\left[y_{n}\,\ln\phi \,\left(w^{T}\,x_{n}+w_{0}\right)+\left(1-y_{n}\right)\,\ln\left(1-\phi \,\left(w^{T}\,x_{n}+w_{0}\right)\right)\right]+\theta \,\left(w,w_{0}\right)$$

This cost-function is minimized on a subset of the data, denoted the training set, and afterward the trained models ability to discriminate is evaluated on a held-out subset, denoted the test set.

We investigate two different regularization approaches. First, we use a regularized logistic regression model where θ(*w*,*w*_0_) is a combination of total variation (TV) and a sparsity prior (L1) (Baldassarre et al., 2012; Dohmatob et al., 2014). The TV-penalty promotes spatial smoothness of the solution, whereas the L1-penalty promotes parsimonious solutions. This model is implemented in the decoding module in the Python package *Nilearn* (Abraham et al., 2014) under the name *SpaceNet.* The second approach we test is a logistic regression with L2-penalty following a dimensionality reduction using principal component analysis (PCA), denoted *pcalogreg* in the results section. We choose to retain 95% of the explained variance in PCA analysis. In both regularization methods, we tune the regularization strength using 5-fold cross-validation in a nested fashion to avoid circularity bias.

For comparison, we also used the Shen parcellation as a way to generate our features for the machine learning analysis. We use a standard logistic regression with L2-penalty (denoted *shen-logreg*) and a linear support vector machine using the standard parameters from *scikit-learn* (denoted *shen-svm).*

### Evaluation Metrics

For both regularization approaches we use repeated (10 times) 5-fold stratified cross-validation, as suggested as a better practice compared to the leave-one-subject-out procedure in Varoquaux et al. (2017). This estimates the generalization accuracy and the area under the receiver operation characteristic (AUC-ROC) of the methods. The ROC is a curve that describes performance of a binary classifier that gives a score to each datum. The score is thresholded to yield a decision of which class to put the particular datum in. In the case of logistic regression described above the scores are the evaluation of ϕ(*w*^*T*^*x*_*n*_ + *w*_0_) and the natural threshold is 0.5, i.e., the datum *x*_*n*_ is classified as class 1 if the probability of class 1 exceeds 0.5. The ROC relates the true positive rate (TPR) to the false positive rate as a function of the threshold applied to the scoring function of the classifier. Integrating the ROC over all the thresholds yields the area-under-the-ROC, i.e., AUC-ROC. Compared to accuracy, it measures how data points are ranked according to the scoring function and not the actual value of the scores. The interpretation of the AUC-ROC is the probability that a randomly datum from class 1 is ranked higher than a randomly selected datum from class 0. Other metrics to assess classifier performance exist such as sensitivity, specificity and F1-score. We opted to use classification accuracy as it is easily interpretable, and the AUC-ROC due to the invariance toward class imbalance.

### Validation of the Machine Learning Models

**FIGURE 1:**
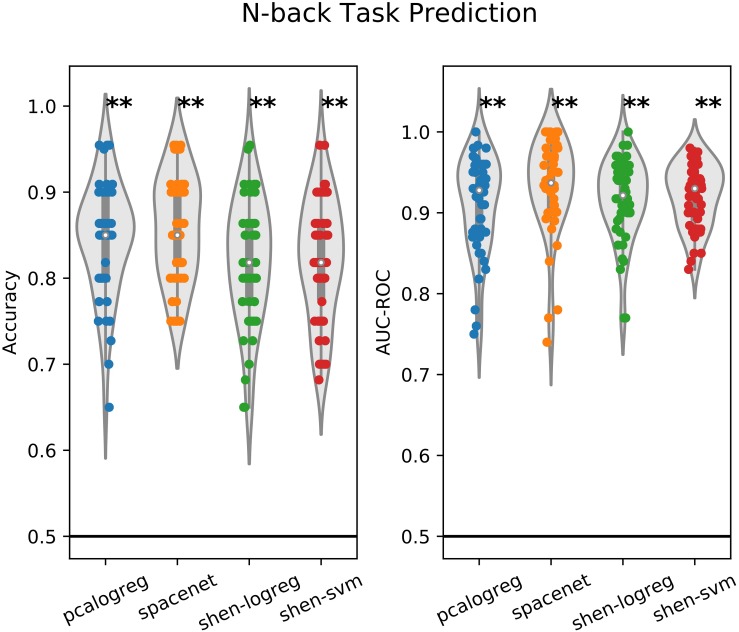
Performance plot of the different classifiers on the working-memory task-response classification (2v0 vs. 2v1 contrast). Two different evaluation measures are used, namely classification accuracy and area-under the receiver operating characteristic (AUC-ROC). The classifiers were evaluated using repeated (10 times) 5-fold cross-validation and each dot in the plot corresponds to the performance measured on one test set. The solid black line indicates the reference classifier of always predicting the largest class in the training set. One-sided *t*-test was used to test if the distribution of the performance over all folds was equal to or worse than the reference classifier (^∗^*p* < 0.05, ^∗∗^*p* < 0.01).

To validate that our machine learning models work, we first evaluated their ability to discriminate the neural response from different task-contrasts within the WM task. We extracted the 2-back vs. 0-back and the 2-back vs. 1-back contrast maps from all participants from the follow-up scanning session and trained the machine learning models distinguish between the two contrast maps across participants. We evaluated the classification using repeated stratified cross-validation (Varoquaux et al., 2017). The performance of the two classifiers for each test-set (*N*_*reps*_ × *N*_*folds*_ = 10 × 5 = 50) is plotted in Figure 1. As a reference model, we compared to the simplest possible model, namely the one that always predicts the largest class in the training set. This is equivalent to random guessing if the class proportions are balanced. This *reference model* was also used in the experiments on real data.

All classifiers performed significantly better than the reference model over the test folds. The sparse model (*SpaceNet*) had a slight advantage over the standard PCA model; however, we did not test this statistically as this was not in the scope of this analysis.

## Results

### Participant Flow and Characteristics

**FIGURE A1:**
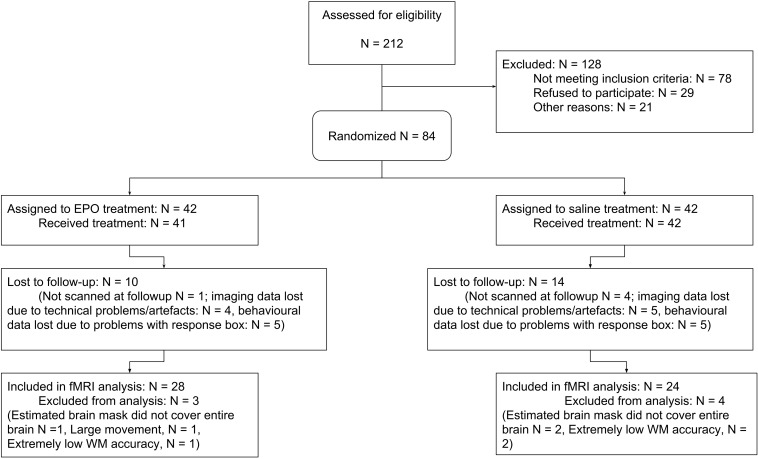
CONSORT diagram for n-back task.

**FIGURE A2:**
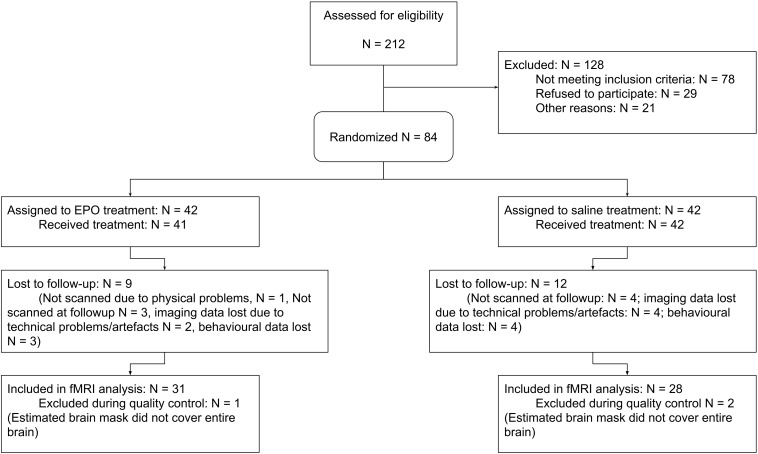
CONSORT diagram for the picture encoding task.

The demographic and clinical characteristics of participants included in the analyses of the two fMRI paradigms are displayed in Tables 1, 2, respectively. The tables were created using the Python package *tableone* (Pollard et al., 2018). CONSORT diagrams for participant flow from inclusion to analysis can be seen in the Appendix (Appendix Figures A1, A2). For details on reasons for exclusions and dropouts see Miskowiak et al. (2016a, b). In addition to the excluded participants in previously published reports (Miskowiak et al., 2016a, b), three participants were excluded after inspecting the brain mask from the first-level analysis as they did not cover the entire brain. Consequently, data were included for 52 participants in the n-back task (EPO: *n* = 28, Saline: *n* = 24) and 59 participants in the picture encoding task (EPO: *n* = 31, Saline: *n* = 28).

**TABLE 1:** Demographics and clinical characteristics for patients included in analysis of n-back task.

**Variable**	**Level**	**Saline (Group = 1)**	**EPO (Group = 2)**	***P*-value**
n		24	28	
Diagnosis, *n* (%)	UD	9 (37.5)	13 (46.4)	0.713
	BD	15 (62.5)	15 (53.6)	
Gender, *n* (%)	Male	10 (41.7)	8 (28.6)	0.486
	Female	14 (58.3)	20 (71.4)	
Age, mean (sd)		40.5 (12.0)	38.5 (9.7)	0.518
Education, mean (sd)		14.9 (2.5)	15.2 (4.2)	0.749
BDI Baseline, mean (sd)		20.7 (10.2)	27.5 (11.5)	0.030
BDI Followup, mean (sd)		18.6 (12.7)	20.3 (12.1)	0.626
HAMD Baseline, mean (sd)		11.6 (6.8)	14.8 (6.7)	0.099
HAMD Followup, mean (sd)		9.7 (7.3)	11.3 (6.3)	0.414
YMRS Baseline, mean (sd)		2.1 (1.9)	2.1 (1.6)	0.917
YMRS Followup, mean (sd)		2.1 (2.7)	1.7 (2.2)	0.651
No. Prior depressions, mean (sd)		5.5 (3.7)	6.3 (4.6)	0.537
No. Previous (hypo)mania, mean (sd)		3.7 (2.3)	5.1 (6.6)	0.470
Bipolar subtype, *n* (%)	1	7 (29.2)	5 (17.9)	0.612
	2	8 (33.3)	10 (35.7)	
	NA	9 (37.5)	13 (46.4)	
Lithium, *n* (%)	0	17 (70.8)	19 (67.9)	0.945
	1	7 (29.2)	9 (32.1)	
Anticonvulsants, *n* (%)	0	12 (50.0)	12 (42.9)	0.597
	1	12 (50.0)	15 (53.6)	
	2		1 (3.6)	
Antidepressants, *n* (%)	0	11 (45.8)	11 (39.3)	0.458
	1	12 (50.0)	17 (60.7)	
	2	1 (4.2)		
Antipsychotics, *n* (%)	0	16 (66.7)	19 (67.9)	0.837
	1	8 (33.3)	9 (32.1)	
Benzodiazepines, *n* (%)	0	17 (70.8)	22 (78.6)	0.205
	1	7 (29.2)	4 (14.3)	
	2		2 (7.1)	
Melatonin, *n* (%)	0	22 (91.7)	22 (78.6)	0.262
	1	2 (8.3)	6 (21.4)	
No medication, *n* (%)	0	23 (95.8)	28 (100.0)	0.462
	1	1 (4.2)		
Number of medications, mean (sd)		2.1 (1.1)	2.4 (1.2)	0.400

*EPO, Erythropoietin; UD, Unipolar disease; BD, bipolar disease; SD, standard deviation; HAMD, Hamilton Depression Rating Scale; BDI, Beck Depression Inventory; and YMRS, Young Mania Rating Scale.*

**TABLE 2:** Demographics and clinical characteristics for participants included in analysis of picture encoding task.

**Variable**	**Level**	**Saline (Group = 1)**	**EPO (Group = 2)**	***P*-value**
n		28	31	
Diagnosis, *n* (%)	UD	13 (46.4)	13 (41.9)	0.933
	BD	15 (53.6)	18 (58.1)	
Gender, *n* (%)	Male	9 (32.1)	10 (32.3)	0.788
	Female	19 (67.9)	21 (67.7)	
Age, mean (sd)		42.8 (11.9)	39.9 (10.7)	0.328
Education, mean (sd)		14.3 (3.0)	15.0 (4.0)	0.418
BDI Baseline, mean (sd)		24.0 (12.0)	25.8 (11.9)	0.572
BDI Followup, mean (sd)		21.1 (13.7)	18.7 (11.7)	0.480
HAMD Baseline, mean (sd)		12.9 (7.2)	14.0 (7.0)	0.564
HAMD Followup, mean (sd)		11.2 (7.2)	10.6 (6.2)	0.697
YMRS Baseline, mean (sd)		2.1 (1.9)	2.6 (3.0)	0.532
YMRS Followup, mean (sd)		1.8 (2.4)	2.1 (2.6)	0.720
No. Prior depressions, mean (sd)		5.6 (4.0)	6.7 (5.4)	0.370
No. Previous (hypo)mania, mean (sd)		4.4 (3.7)	4.9 (6.0)	0.778
Bipolar subtype, *n* (%)	1	7 (25.0)	7 (22.6)	0.851
	2	8 (28.6)	11 (35.5)	
	NA	13 (46.4)	13 (41.9)	
Lithium, *n* (%)	0	21 (75.0)	22 (71.0)	0.956
	1	7 (25.0)	9 (29.0)	
Anticonvulsants, *n* (%)	0	16 (57.1)	14 (45.2)	0.313
	1	12 (42.9)	15 (48.4)	
	2		2 (6.5)	
Antidepressants, *n* (%)	0	10 (35.7)	11 (35.5)	0.565
	1	17 (60.7)	20 (64.5)	
	2	1 (3.6)		
Antipsychotics, *n* (%)	0	19 (67.9)	23 (74.2)	0.804
	1	9 (32.1)	8 (25.8)	
Benzodiazepines, *n* (%)	0	17 (60.7)	24 (77.4)	0.218
	1	10 (35.7)	5 (16.1)	
	2	1 (3.6)	2 (6.5)	
Melatonin, *n* (%)	0	25 (89.3)	25 (80.6)	0.477
	1	3 (10.7)	6 (19.4)	
No medication, *n* (%)	0	27 (96.4)	31 (100.0)	0.475
	1	1 (3.6)		
Number of medications, mean (sd)		2.2 (1.2)	2.3 (1.2)	0.808

*EPO, erythropoietin; UD, unipolar disease; BD, bipolar disease; SD, standard deviation; HAMD, Hamilton Depression Rating Scale; BDI, Beck Depression Inventory; and YMRS, Young Mania Rating Scale.*

### Prediction Based on the Spatial Working Memory Paradigm

**FIGURE 2:**
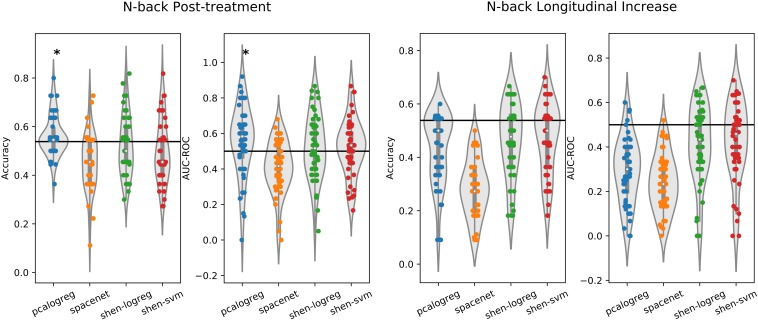
Performance plot of the different classifiers on the working-memory data, both cross-sectional after followup (left plot) and longitudinal increase from baseline to followup (right plot). The classifiers were trained to distinguish participants by pharmacological treatment (EPO vs. Saline). Two different evaluation measures are used, namely classification accuracy and area-under the receiver operating characteristic (AUC-ROC). The classifiers were evaluated using repeated (10 times) 5-fold cross-validation and each dot in the plot corresponds to the performance measured on one test set. The solid black line indicates the reference classifier of always predicting the largest class in the training set. One-sided *t*-test was used to test if the distribution of the performance over all folds was equal to or worse than the reference classifier (^∗^*p* < 0.05).

Results from the prediction of the pharmacological treatment (EPO vs. Saline) from subject–level 2-back vs. 0-back contrast maps can be seen in Figure 2 and in the performance summary in Table 3. In contrast with our hypothesis, the supervised machine learning models were unable to produce a robust prediction of whom had received EPO (vs. saline) based on the whole-brain task-related fMRI data from the post-treatment scan. However, the ‘pcalogreg’ model, reflecting non-sparse distributed patterns of activity, was able to predict EPO group membership better than the reference classifier, i.e., always predicting the largest class. Using the ‘pcalogreg’ model, we obtained a mean accuracy over folds of about 56%, however, there was a large variability over folds with a portion of test folds falling below the reference model, equivalent to random guessing. We found similar low prediction performance of the classifiers in both cross-sectional and the longitudinal data sets. The ‘pcalogreg’ model was not able to achieve better classification than the reference when utilizing baseline data, which could point toward cross-sectional n-back data being more informative for predicting pharmacological treatment. We compared the two whole-brain approaches (*spacenet* and *pcalogreg)* to using the Shen parcellation (*shen-logreg* and *shen-svm*), which produced comparable performance.

**TABLE 3:** Summary of all analyses.

	**Accuracy**	**AUC-ROC**
**N-back Cross-sectional**		
*pcalogreg*	0.5617^∗^	0.5541^∗^
*spacenet*	0.4523	0.4099
*shen-logreg*	0.5212	0.5211
*shen-svm*	0.4986	0.5190
**N-back Longitudinal Increase**		
*pcalogreg*	0.4336	0.2951
*spacenet*	0.2859	0.2411
*shen-logreg*	0.4657	0.4157
*shen-svm*	0.4697	0.4229
**Picture Encoding Cross-sectional**		
*pcalogreg*	0.4694	0.4031
*spacenet*	0.5178	0.5472^∗^
*shen-logreg*	0.4750	0.4878
*shen-svm*	0.4433	0.4423
**Picture Encoding Longitudinal Increase**		
*pcalogreg*	0.4944	0.4408
*spacenet*	0.5898^∗∗^	0.5989^∗∗^
*shen-logreg*	0.4501	0.4836
*shen-svm*	0.3953	0.3826

*We report the performance (accuracy and AUC-ROC) of each of the classifiers applied to all the data sets. Classifier performance was assessed using repeated 5-fold cross-validation, and the value reported is the mean over all 50-folds. One-sided *t*-test was used to test if the distribution of the performance over all folds was equal to or worse than the reference classifier (^∗^*p* < 0.05, ^∗∗^*p* < 0.01).*

### Prediction Based on the Explicit Picture Encoding Paradigm

**FIGURE 3:**
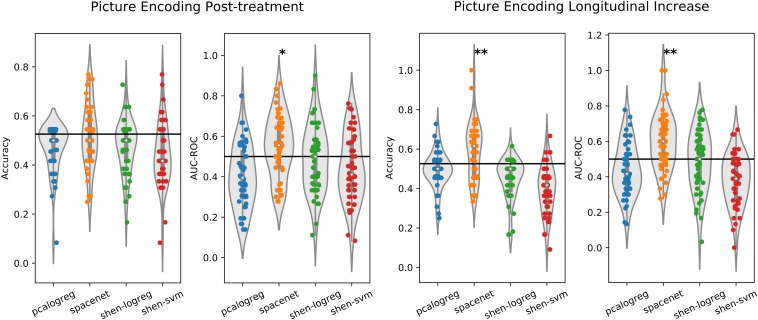
Performance plot of the different classifiers on the picture encoding data, both cross-sectional after followup (left plot) and longitudinal increase from baseline to followup (right plot). The classifiers were trained to distinguish participants by pharmacological treatment (EPO vs. Saline). Two different evaluation measures are used, namely classification accuracy and area-under the receiver operating characteristic (AUC-ROC). The classifiers were evaluated using repeated (10 times) 5-fold cross-validation and each dot in the plot corresponds to the performance measured on one test set. The solid black line indicates the reference classifier of always predicting the largest class in the training set. One-sided *t*-test was used to test if the distribution of the performance over all folds was equal to or worse than the reference classifier (^∗^*p* < 0.05, ^∗∗^*p* < 0.01).

Results from the supervised machine learning prediction of treatment status based on the picture encoding data can be seen in Figure 3 and in the performance summary in Table 3. Pertaining to our main hypothesis, we found no evidence that the pharmacological status could be classified better than the reference model from the post-treatment brain response as measured by classification accuracy. Regarding the difference between cross-sectional and longitudinal analysis, the *SpaceNet* classifier, reflecting highly localized features of the brain response, was able to achieve significantly better than random guessing in longitudinal data. However, as with the n-back data there was quite a large variability over test folds and the estimated generalization performance did not exceed chance level by a large margin (around 59% mean accuracy). As with the WM paradigm, we also did the analysis using the Shen parcellation and found similar results, as shown in Figure 3 and Table 3.

## Discussion

In this exploratory fMRI report based on our published EPO RCTs (Miskowiak et al., 2014a, b), we applied a supervised machine learning algorithm to explore the EPO-associated changes in neural activity during spatial WM and picture encoding in a sample (*n* = 56–59) of patients with mood disorders. Complementary to the classical hypothesis testing approach already presented in previously published papers, we here used a supervised machine learning method in line with (Barron et al., 2018) using COPES as input to the algorithm. In contrast with our hypothesis, we found no task-related patterns of neural activity that could predict held-out participants treatment status at a reliable level of performance (≤60% accuracy). However, in some of the cases, the algorithms performed significantly better than random guessing, albeit with classification accuracies reaching only around 60% and with large variability over cross-validation folds. Further, comparison of the performance of the machine learning algorithms applied to cross-sectional post-treatment data sets and longitudinal (follow-up minus baseline) data sets showed comparable (low) performance.

The supervised multivariate machine learning algorithms failed to predict which participants had received EPO (vs. placebo) with high reliability. In the cases where the models performed significantly better than chance, the accuracy was low, with only 60% of participants being correctly classified as having received EPO. As a sanity check, we therefore took a step back and trained the two machine learning models on the easier problem of distinguishing contrasts from different experimental conditions in the spatial n-back WM data follow-up data. This step was successful in terms of classification accuracy, which in the case of the *SpaceNet* model reached an average of over 80% over test folds. This model validation step gave an indication of what classification performance that could be expected in a best-case scenario, indicating that the models were trainable in this high-dimensional problem. Nevertheless, the poor predictive value of machine learning algorithms in characterizing the pharmacological effects on neural activity using task-related fMRI paradigms aligns well with the observations in a recent machine learning study of the profile of pharmacological effects on neural activity (Barron et al., 2018). Specifically, we observed comparable performance in terms of within-study classification accuracy, however on a larger sample size than each of the individual studies analyzed in Barron et al. (2018).

The machine learning models used were statistically tested using a *t*-test on the scores obtained from the cross-validation procedure against a reference model, that always predicted the largest class in the training set. We acknowledge that this is not the best way to test the significance of the classification performance, and one should optimally have used permutation testing (Ojala and Garriga, 2010). However, due to computational complexity of the SpaceNet classifier this was not feasible. We do not expect this to dramatically change the results, due to the poor performance of the classifiers.

As pointed out by Barron et al. (2018), inter-participant variability stemming from differences in brain anatomy and functional localization is a confounding source for the classification results obtained. One way to address this problem would be to have a cross-over design in which each participant would at some point get both treatments and would thus serve as its own placebo control. This was suggested by Barron et al. (2018) following results from a pain medication efficacy study (Duff et al., 2015). We used the difference in the neural response to task from baseline to follow-up to eliminate some of the inter-participant variability. However, our data suggests that the longitudinal approach does not sufficiently deals with this problem as we observed no consistent differences in the predictive power of the machine learning models when applied to the longitudinal versus the cross-sectional and longitudinal data sets. One possible explanation for this is the large *intra-participant* variability stemming from motion effects (Lund et al., 2005) and day-to-day variation from caffeine and food consumption (Poldrack et al., 2015), that could mask the true pharmacological difference effect between baseline and follow-up. Disentangling intra- and inter-participant variability is an active area of research that needs further investigation (Gordon et al., 2017).

The fMRI data from the EPO trials have already been analyzed for the picture encoding task in Miskowiak et al. (2016a) and for the n-back task in Miskowiak et al. (2016b). The poor classification results were somewhat surprising given the moderate to large effect sizes of EPO versus placebo on hippocampus-dependent memory and global cognition across these patients (Miskowiak et al., 2015; Ott et al., 2016) and our observation of EPO-associated changes in dorsal prefrontal and parietal activity during the same fMRI paradigms in the hypothesis-driven general linear modeling analyses (Miskowiak et al., 2016a, b). One could therefore ponder why the significant neuronal activity differences induced by pharmacological treatment found in the previous papers are not in the same way reflected in the results of the present machine learning analyses. We underline here that there are large methodological differences that relate to the difference between encoding and decoding in neuroimaging (Varoquaux and Poldrack, 2018) and the difference between explanation and prediction in psychology (Yarkoni and Westfall, 2017). In the classical statistics approach, we try to come up with mechanistic explanations for the data generating process and use data to estimate the parameters of that process. In contrast, the machine learning approach involves the development of a data-driven algorithm to produce the same predictions as the data generating process which is validated using out-of-sample estimates. These two approaches do therefore not necessarily overlap in their conclusions as illustrated in this report and as pointed out by others (Bzdok et al., 2018); highly significant result (in the classical sense) provide no guarantees on the (held-out) predictive capabilities of the model so it cannot be excluded that a more marked difference between the two groups (e.g., *p*-values ≤ 0.001) would have resulted in a higher accuracy.

The sample size of studies using pharmaco-related fMRI have been rather small, ranging from 12 to 42 patients (Iannetti et al., 2005; Harmer et al., 2006; Murphy et al., 2009; Godlewska et al., 2012; Wanigasekera et al., 2012; Harris et al., 2013; Lee et al., 2013; Di Simplicio et al., 2014; Sanders et al., 2015). Our data set considered 52 participants, resulting in a larger sample size compared to the previous pharmaco-related fMRI studies. Yet the risk of conducting a type II error has still to be considered. The treatment groups were comparable on diagnostic, clinical and demographic variables as well as medication status. Nevertheless, the variability in mood symptoms between patients and medication status may have affected the ability to detect a clear signal in neural activity patterns and thus contributed to the suboptimal performance of the algorithm. Further, we applied the machine learning models to multiple fMRI task paradigms to corroborate what conclusion that can be drawn reliably from different tests. It was a methodological strength that we used two different machine learning methods, including a prominent sparse model targeted for neuroimaging data, namely the SpaceNet classifier (Gramfort et al., 2013). Furthermore, we evaluated our models using repeated cross-validation, which is considered a best practice within performance evaluation for neuroimaging decoders (Varoquaux et al., 2017). However, the gold-standard of evaluating the classifiers on a completely held-out sample, as done in Browning et al. (2018), was not feasible. A limitation was that the multivariate decoding methods used estimate many more parameters compared to the number of observations. Thus, regularization approaches are needed to control overfitting, i.e., finding patterns that do not generalize to a new population. In Barron et al. (2018) this was achieved by mapping the participant level COPE values into a resting-state based parcellation from Shen et al. (2013) with 268 parcels. We compared our approach to using a parcellation and found similar performance across all tasks. We acknowledge that the search for the optimal regularization strength in logistic regression is a difficult problem and has a large influence on the final classification performance. However, since our models were able to reasonably well predict the spatial n-back WM task contrasts in the validation experiment, the choice of regularization strength is unlikely to be the source of the relatively poor classification performance.

## Conclusion

In conclusion, we found no reliable evidence that pharmacological treatment with EPO could be predicted from neural signatures extracted from task-based fMRI with a supervised machine learning algorithm. While one of the algorithms was able to predict treatment status significantly better than the reference model, the prediction accuracy was relatively low (≈60% accuracy). This result suggests that we need larger sample sizes to detect whole-brain patterns of activity that can predict the administration of EPO and other potential precognitive interventions.

## Data Availability Statement

The datasets for this manuscript are not publicly available because the data contains sensitive information about medication, medical diagnosis and other clinical variables. Requests to access the datasets should be directed to KWM, kamilla.woznica.miskowiak@regionh.dk.

## Author Contributions

SN, KHM, and KWM designed the study and wrote the analysis plan. KWM, MV, LK, and HS designed the original fMRI studies. SN wrote and ran the necessary code for the machine learning analysis. SN and KWM wrote the first draft of the manuscript. All authors contributed to and have approved the final manuscript.

## Conflict of Interest

KWM reports having received consultancy fees from Allergan and Janssen within the past 3 years. MV discloses consultancy fees from Lundbeck and AstraZeneca within the last 3 years. HS discloses honoraria as reviewing Editor for Neuroimage, as speaker for Biogen Idec Denmark A/S, and scientific advisor for Lundbeck within the past 3 years. LK reports having been a consultant for Lundbeck, AstraZeneca and Sunovion within the last 3 years. The remaining authors declare that the research was conducted in the absence of any commercial or financial relationships that could be construed as a potential conflict of interest.

## Footnotes

1 http://fsl.fmrib.ox.ac.uk/fsl/fslwiki/
